# Supplementary material for: Construction of PIK3C3 Transgenic Pig and Its Pathogenesis of Liver Damage
Source: Life (Basel). 2022 Apr 24;12(5):630. doi: 10.3390/life12050630 (PMC9146193; doi:10.3390/life12050630)
Supplement: Supplementary file 1 [file life-12-00630-s001.zip › life-1592413-supplementary.pdf]

Supplementary materials 1 pUC57-PIK3C3 sequence

10 20 30 40 50 60

CTCGAGATGGGGGAAGCAGAGAAGTTTCACTACATCTACAGTTGTGACCTGGATATCAACGTGCAG

A6907 PIK3C3.SEQ(1>2676) → Ctcgagatgggggaagcagagaagtttcactacatctacagttgtgacctggatatcaacgtgcag

140419R9088-1R22(A6907)P57F_Z_A07.ab1(1>700) →

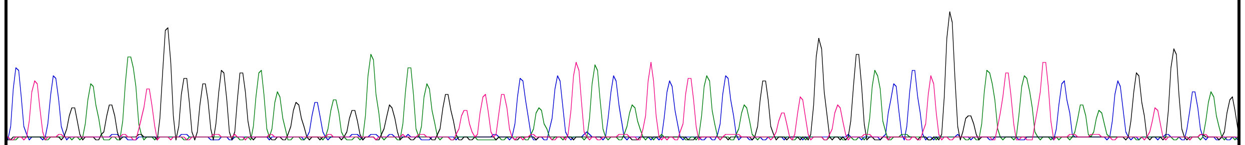

CTCGAGATGGGGGAAGCAGAGAAGTTTCACTACATCTACAGTTGTGACCTGGATATCAACGTGCAG

140419R9088-1R22(A6907)M13+_Z_A01.ab1(1>700) →

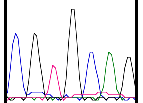

CGTGCAG

70 80 90 100 110 120 130

CTTAAGATAGGAAGTTTGGAAGGGAAGAGAGAACAAAAGAGTTATAAAGCCGTTCTAGAAGACCCA

A6907 PIK3C3.SEQ(1>2676) → cttaagataggaagtttggaagggaagagagaacaaaagagttataaagccgttctagaagaccca

140419R9088-1R22(A6907)P57F_Z_A07.ab1(1>700) →

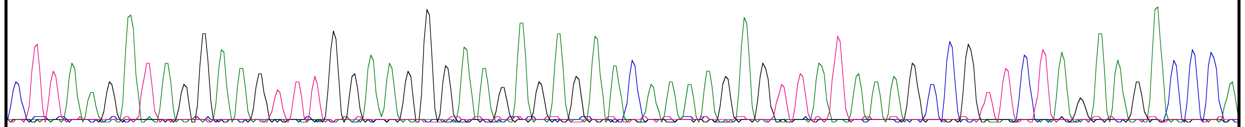

CTTAAGATAGGAAGTTTGGAAGGGAAGAGAGAACAAAAGAGTTATAAAGCCGTTCTAGAAGACCCA

140419R9088-1R22(A6907)M13+_Z_A01.ab1(1>700) →

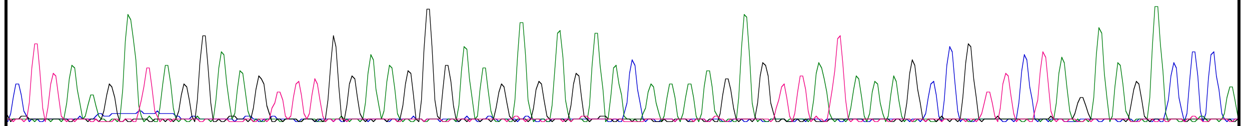

CTTAAGATAGGAAGTTTGGAAGGGAAGAGAGAACAAAAGAGTTATAAAGCCGTTCTAGAAGACCCA

140 150 160 170 180 190

ATGTTGAAGTTTTCAGGACTATACCAAGAAACATGTTCTGACCTCTATGTCACTTGCCAAGTTTTT

A6907 PIK3C3.SEQ(1>2676) → atgttgaagttttcaggactataccaagaaacatgttctgacctctatgtcacttgccaagttttt

140 150 160 170 180 190

ATGTTGAAGTTTTCAGGACTATACCAAGAAACATGTTCTGACCTCTATGTCACTTGCCAAGTTTTT

140419R9088-1R22(A6907)P57F_Z_A07.ab1(1>700) →

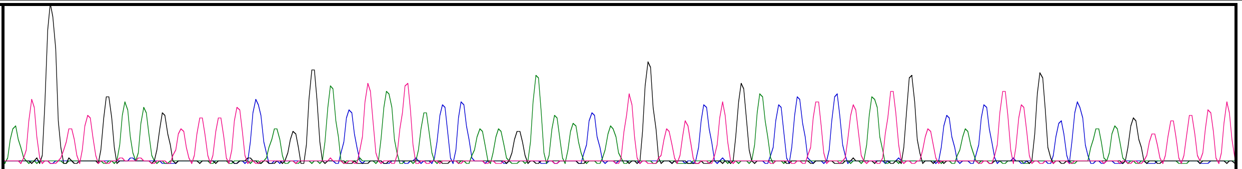

ATGTTGAAGTTTTCAGGACTATACCAAGAAACATGTTCTGACCTCTATGTCACTTGCCAAGTTTTT

140419R9088-1R22(A6907)M13+_Z_A01.ab1(1>700) →

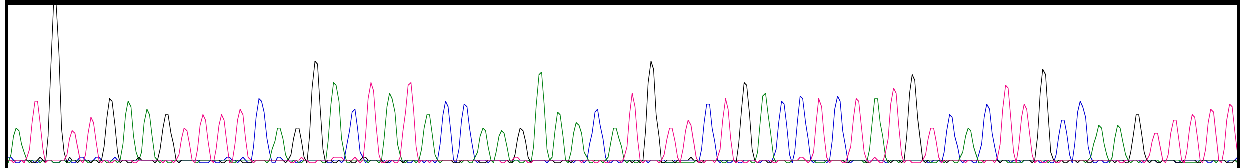

ATGTTGAAGTTTTCAGGACTATACCAAGAAACATGTTCTGACCTCTATGTCACTTGCCAAGTTTTT

200 210 220 230 240 250 260

GCAGAAGGAAAGCCTCTGGCCTTGCCAGTAAGAACTTCGTATAAGGCATTTAGCACAAGATGGAAC

A6907 PIK3C3.SEQ(1>2676) → gcagaaggaaagcctctggccttgccagtaagaacttcgtataaggcatttagcacaagatggaac

140419R9088-1R22(A6907)P57F_Z_A07.ab1(1>700) →

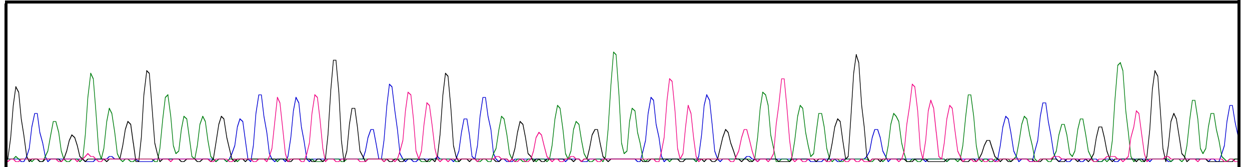

GCAGAAGGAAAGCCTCTGGCCTTGCCAGTAAGAACTTCGTATAAGGCATTTAGCACAAGATGGAAC

140419R9088-1R22(A6907)M13+_Z_A01.ab1(1>700) →

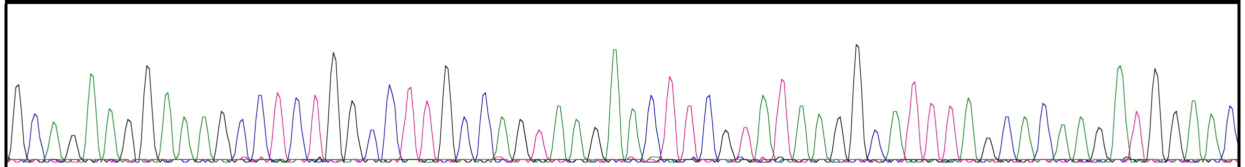

GCAGAAGGAAAGCCTCTGGCCTTGCCAGTAAGAACTTCGTATAAGGCATTTAGCACAAGATGGAAC

270 280 290 300 310 320 330

TGGAATGAATGGCTGAAACTGCCTGTGAAATACCCTGACCTGCCCAGGAATGCCCAAGTGGCCCTG

A6907 PIK3C3.SEQ(1>2676) → tggaatgaatggctgaaactgcctgtgaaataccctgacctgcccaggaatgcccaagtggccctg

140419R9088-1R22(A6907)P57F_Z_A07.ab1(1>700) →

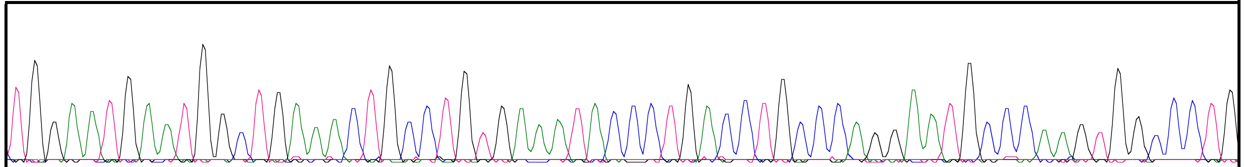

TGGAATGAATGGCTGAAACTGCCTGTGAAATACCCTGACCTGCCCAGGAATGCCCAAGTGGCCCTG

270 280 290 300 310 320 330

TGGAATGAATGGCTGAAACTGCCTGTGAAATACCCTGACCTGCCCAGGAATGCCCAAGTGGCCCTG

140419R9088-1R22(A6907)M13+_Z_A01.ab1(1>700) →

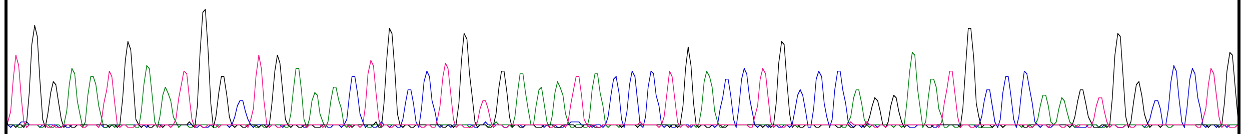

TGGAATGAATGGCTGAAACTGCCTGTGAAATACCCTGACCTGCCCAGGAATGCCCAAGTGGCCCTG

340 350 360 370 380 390

ACTATATGGGATGTCTATGGACCAGGAAAGGCAGTGCCTGTGGGTGGAACAACAGTTTCATTGTTT

A6907 PIK3C3.SEQ(1>2676) → actatatgggatgtctatggaccaggaaaggcagtgcctgtgggtggaacaacagtttcattgttt

140419R9088-1R22(A6907)P57F_Z_A07.ab1(1>700) →

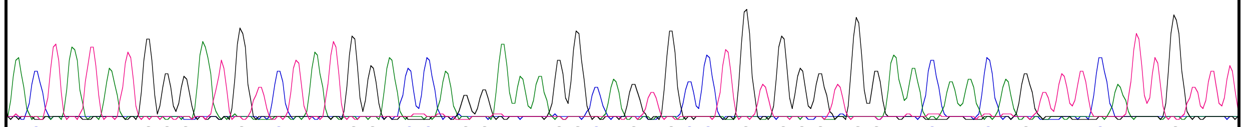

ACTATATGGGATGTCTATGGACCAGGAAAGGCAGTGCCTGTGGGTGGAACAACAGTTTCATTGTTT

140419R9088-1R22(A6907)M13+_Z_A01.ab1(1>700) →

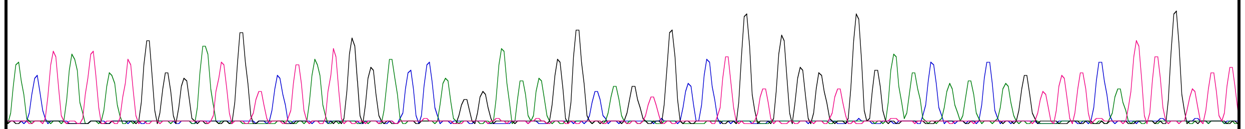

ACTATATGGGATGTCTATGGACCAGGAAAGGCAGTGCCTGTGGGTGGAACAACAGTTTCATTGTTT

400 410 420 430 440 450 460

GGAAAATACGGCATGTTTCGCCAAGGGATGCATGACTTGAAAGTCTGGCCTAATGTGGAAGCAGAC

A6907 PIK3C3.SEQ(1>2676) → ggaaaatacggcatgtttcgccaagggatgcatgacttgaaagtctggcctaatgtggaagcagac

140419R9088-1R22(A6907)P57F_Z_A07.ab1(1>700) →

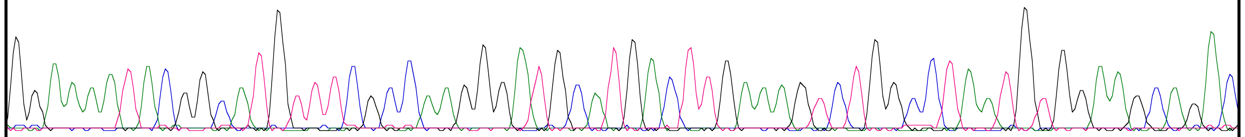

GGAAAATACGGCATGTTTCGCCAAGGGATGCATGACTTGAAAGTCTGGCCTAATGTGGAAGCAGAC

140419R9088-1R22(A6907)M13+_Z_A01.ab1(1>700) →

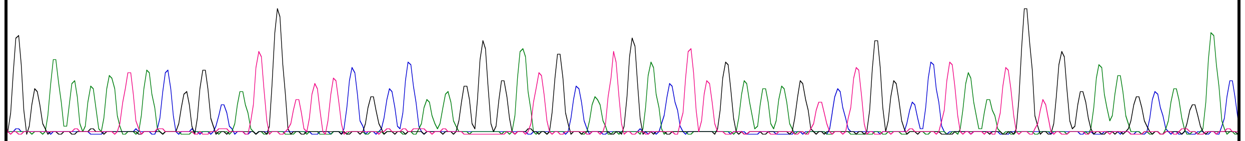

GGAAAATACGGCATGTTTCGCCAAGGGATGCATGACTTGAAAGTCTGGCCTAATGTGGAAGCAGAC

470 480 490 500 510 520

GGATCAGAACCCACAAAAACTCCTGGCAGAACAAGCAGTACTCTCTCAGAAGATCAGATGAGCCGT

A6907 PIK3C3.SEQ(1>2676) → ggatcagaacccacaaaaactcctggcagaacaagcagtactctctcagaagatcagatgagccgt

140419R9088-1R22(A6907)P57F_Z_A07.ab1(1>700) →

140419R9088-1R22(A6907)M13+_Z_A01.ab1(1>700) →

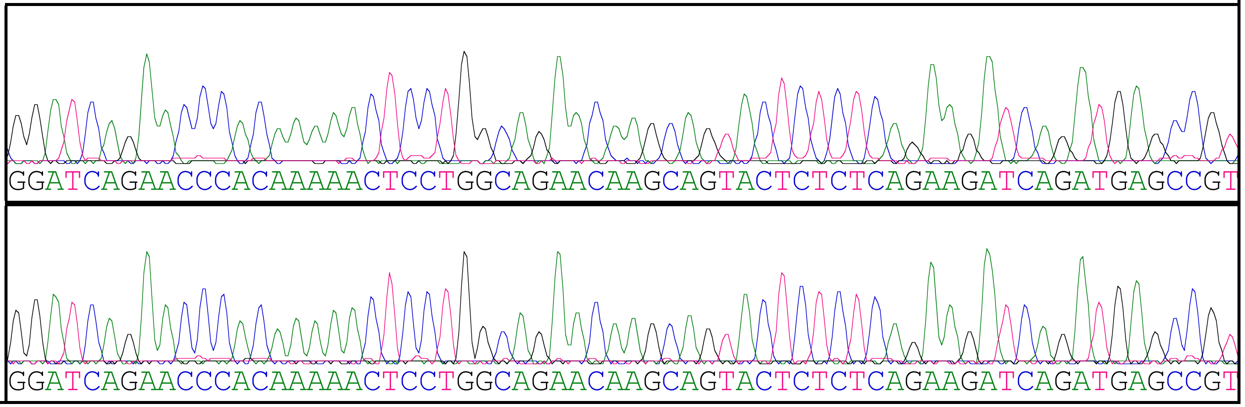

530 540 550 560 570 580 590

CTTGCCAAGCTCACCAAGGCTCATCGACAAGGTCACATGGTGAAAGTAGATTGGCTGGACAGACTG

A6907 PIK3C3.SEQ(1>2676) → cttgccaagctcaccaaggctcatcgacaaggtcacatggtgaaagtagattggctggacagactg

140419R9088-1R22(A6907)P57F_Z_A07.ab1(1>700) →

140419R9088-1R22(A6907)M13+_Z_A01.ab1(1>700) →

140419R9088-1R22(A6907)A6907F1_Z_C01.ab1(1>700) →

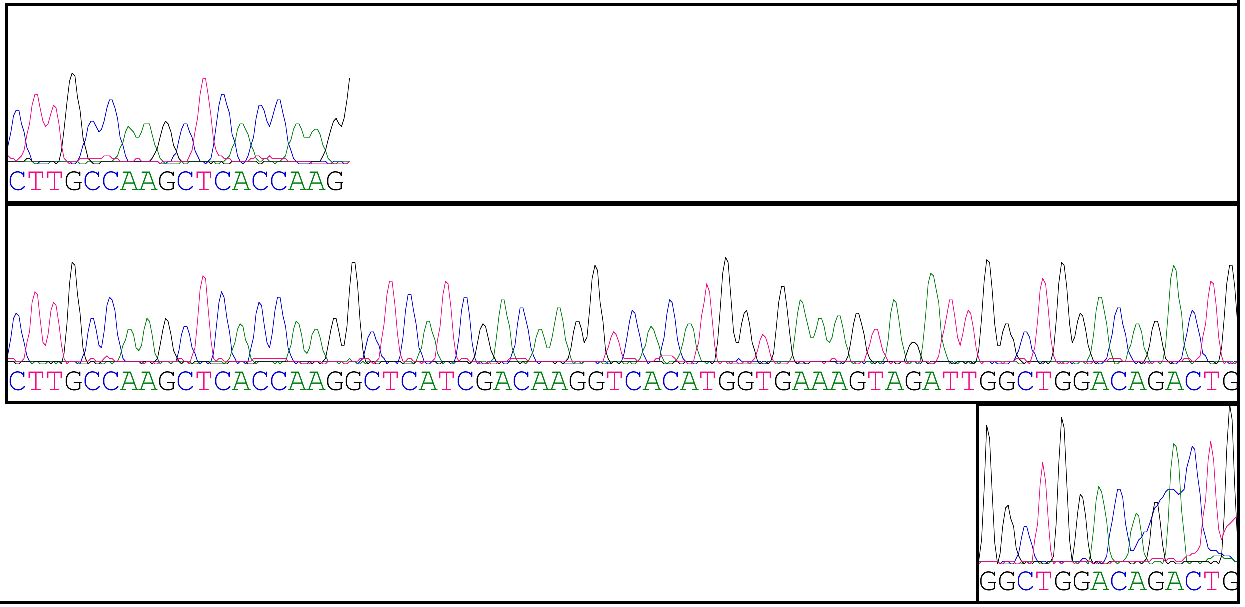

600 610 620 630 640 650 660

ACCTTTAGAGAAATAGAAATGATAAATGAGAGTGAAAAACGAAGTTCTAATTTCATGTACTTGATG

A6907 PIK3C3.SEQ(1>2676) → acctttagagaaatagaaatgataaatgagagtgaaaaacgaagttctaatttcatgtacttgatg

140419R9088-1R22(A6907)M13+_Z_A01.ab1(1>700) →

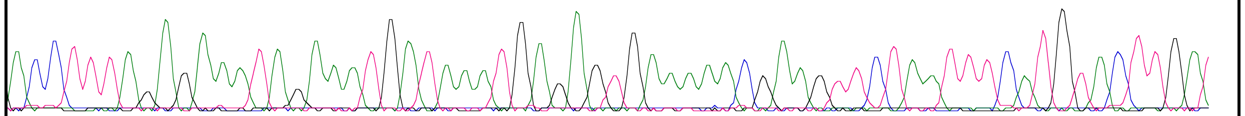

ACCTTTAGAGAAATAGAAATGATAAATGAGAGTGAAAAACGAAGTTCTAATTTCATGTACTTGA

140419R9088-1R22(A6907)A6907F1_Z_C01.ab1(1>700) →

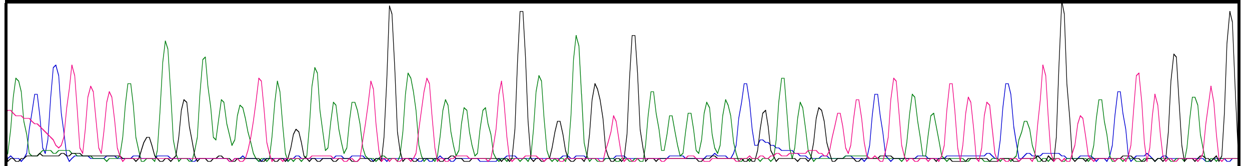

ACCTTTAGAGAAATAGAAATGATAAATGAGAGTGAAAAACGAAGTTCTAATTTCATGTACTTGATG

670 680 690 700 710 720

GTTGAGTTTCGATGTGTCAAGTGTGATGATAAAGAATATGGTATTGTTTATTATGAAAAGGATGGT

A6907 PIK3C3.SEQ(1>2676) → gttgagtttcgatgtgtcaagtgtgatgataaagaatatggtattgtttattatgaaaaggatggt

140419R9088-1R22(A6907)A6907F1_Z_C01.ab1(1>700) →

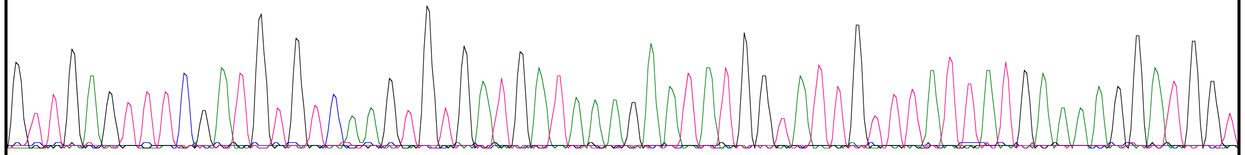

GTTGAGTTTCGATGTGTCAAGTGTGATGATAAAGAATATGGTATTGTTTATTATGAAAAGGATGGT

730 740 750 760 770 780 790

GACGAGTCATCTCCCATTTTAACGGGCTTTGAGATAGTGAAAGTTCCTGACCCTCAAATGTCTATG

A6907 PIK3C3.SEQ(1>2676) → gacgagtcatctcccattttaacgggctttgagatagtgaaagttcctgaccctcaaatgtctatg

140419R9088-1R22(A6907)A6907F1_Z_C01.ab1(1>700) →

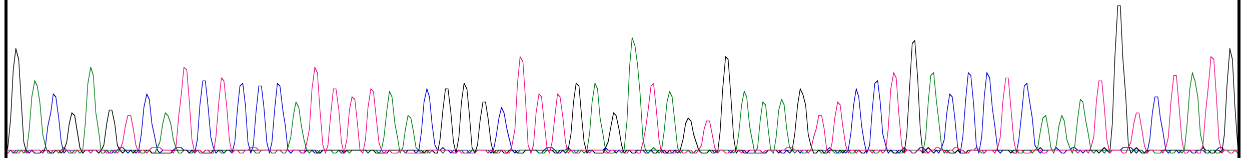

GACGAGTCATCTCCCATTTTAACGGGCTTTGAGATAGTGAAAGTTCCTGACCCTCAAATGTCTATG

800 810 820 830 840 850

GAGAATTTAGTTGAGAGCAAACACCACAAACTTGCTCGGAGTTTAAGAAGTGGACCTTCTGACCAT

A6907 PIK3C3.SEQ(1>2676) → gagaatttagttgagagcaaacaccacaaacttgctcggagtttaagaagtggaccttctgaccat

140419R9088-1R22(A6907)A6907F1_Z_C01.ab1(1>700) →

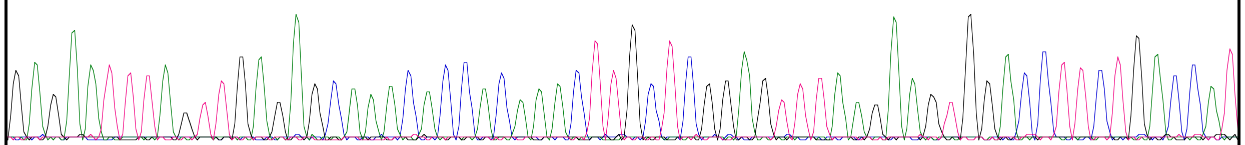

GAGAATTTAGTTGAGAGCAAACACCACAAACTTGCTCGGAGTTTAAGAAGTGGACCTTCTGACCAT

860 870 880 890 900 910 920

GGTCTCAAACCTAATGCTGCCACGAGAGATCAACTGAATATTATTGTGAGTTATCCACCAACCAAG

A6907 PIK3C3.SEQ(1>2676) → ggtctcaaacctaatgctgccacgagagatcaactgaatattattgtgagttatccaccaaccaag

140419R9088-1R22(A6907)A6907F1_Z_C01.ab1(1>700) →

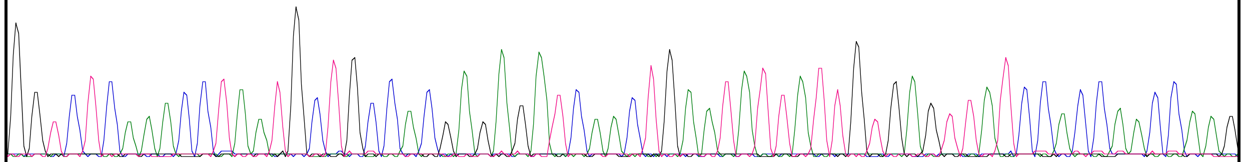

GGTCTCAAACCTAATGCTGCCACGAGAGATCAACTGAATATTATTGTGAGTTATCCACCAACCAAG

930 940 950 960 970 980 990

CAACTTACATATGAAGAACAAGATCTTGTATGGAAGTTTAGATATTATCTTACTAATCAAGAAAAA

A6907 PIK3C3.SEQ(1>2676) → caacttacatatgaagaacaagatcttgtatggaagtttagatattatcttactaatcaagaaaaa

140419R9088-1R22(A6907)A6907F1_Z_C01.ab1(1>700) →

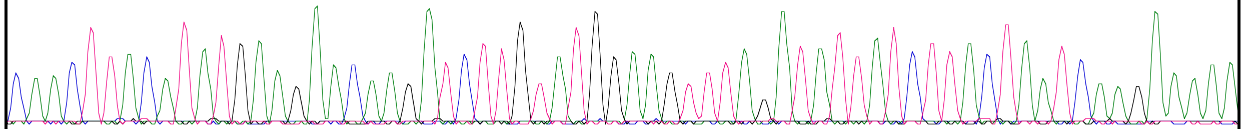

CAACTTACATATGAAGAACAAGATCTTGTATGGAAGTTTAGATATTATCTTACTAATCAAGAAAAA

1000 1010 1020 1030 1040 1050

GCTTTGACAAAGTTCTTGAAATGCGTTAATTGGGATCTACCTCAGGAGGCCAAACAGGCATTGGAA

A6907 PIK3C3.SEQ(1>2676) → gctttgacaaagttcttgaaatgcgttaattgggatctacctcaggaggccaaacaggcattggaa

1000 1010 1020 1030 1040 1050

GCTTTGACAAAGTTCTTGAAATGCGTTAATTGGGATCTACCTCAGGAGGCCAAACAGGCATTGGAA

140419R9088-1R22(A6907)A6907F1_Z_C01.ab1(1>700)→

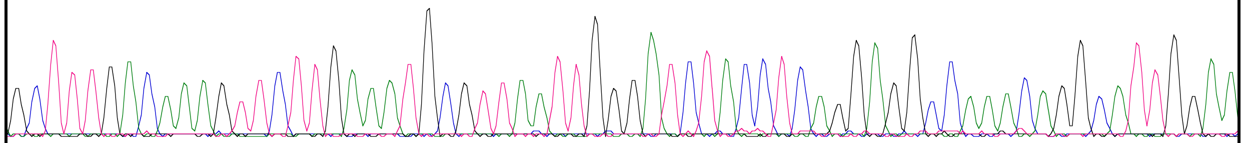

GCTTTGACAAAGTTCTTGAAATGCGTTAATTGGGATCTACCTCAGGAGGCCAAACAGGCATTGGAA

1060 1070 1080 1090 1100 1110 1120

CTTCTGGGAAAATGGAAGCCAATGGATGTAGAGGATTCCTTGGAGCTATTATCATCCCATTACGCC

A6907 PIK3C3.SEQ(1>2676) → cttctgggaaaatggaagccaatggatgtagaggattccttggagctattatcatcccattacgcc

140419R9088-1R22(A6907)A6907F1_Z_C01.ab1(1>700)→

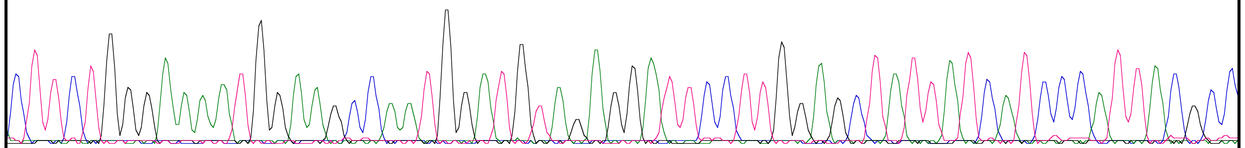

CTTCTGGGAAAATGGAAGCCAATGGATGTAGAGGATTCCTTGGAGCTATTATCATCCCATTACGCC

140419R9088-1R22(A6907)A6907F2_Z_D01.ab1(1>700)→

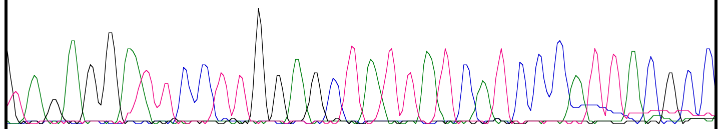

TAGAGGATTCCTTGGAGCTATTATCATCCCATTACGCC

1130 1140 1150 1160 1170 1180

AATCCAACAGTGAGACGTTATGCAGTTGCCCGATTACGGCAGGCAGATGATGAGGATTTGTTGATG

A6907 PIK3C3.SEQ(1>2676) → aatccaacagtgagacgttatgcagttgcccgattacggcaggcagatgatgaggatttgttgatg

140419R9088-1R22(A6907)A6907F1_Z_C01.ab1(1>700)→

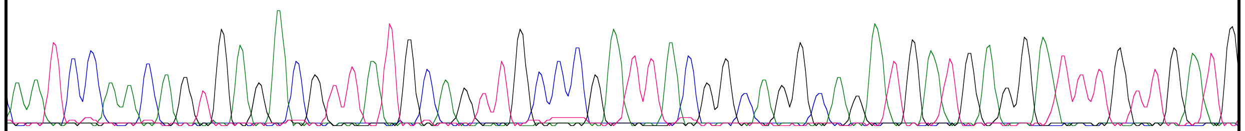

AATCCAACAGTGAGACGTTATGCAGTTGCCCGATTACGGCAGGCAGATGATGAGGATTTGTTGATG

140419R9088-1R22(A6907)A6907F2_Z_D01.ab1(1>700)→

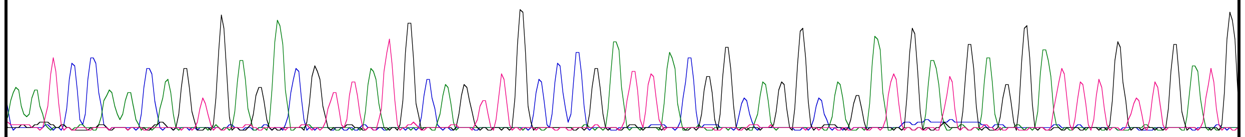

AATCCAACAGTGAGACGTTATGCAGTTGCCCGATTACGGCAGGCAGATGATGAGGATTTGTTGATG

1190 1200 1210 1220 1230 1240 1250

TACCTATTACAGCTCGTCCAAGCTCTCAAATATGAAAATTTTGATGACATAAAGAATGGATTAGAA

A6907 PIK3C3.SEQ(1>2676) → tacctattacagctcgtccaagctctcaaatatgaaaattttgatgacataaagaatggattagaa

140419R9088-1R22(A6907)A6907F1_Z_C01.ab1(1>700) →

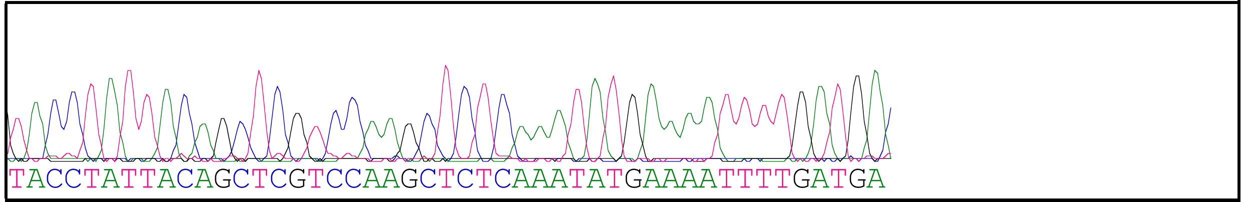

140419R9088-1R22(A6907)A6907F2_Z_D01.ab1(1>700) →

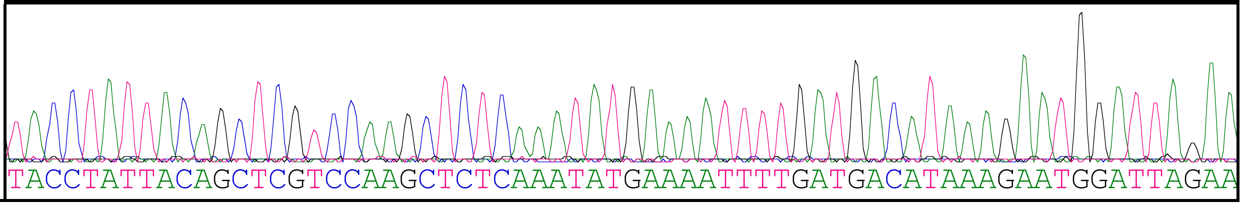

1260 1270 1280 1290 1300 1310 1320

CCTACCAAGAAGGAAAGTCAGGGTTCAGTGTCAGAGAGTGTGTCAAATTCTGGAATAGGTTCTGCA

A6907 PIK3C3.SEQ(1>2676) → cctaccaagaaggaaagtcagggttcagtgtcagagagtgtgtcaaattctggaataggttctgca

140419R9088-1R22(A6907)A6907F2_Z_D01.ab1(1>700) →

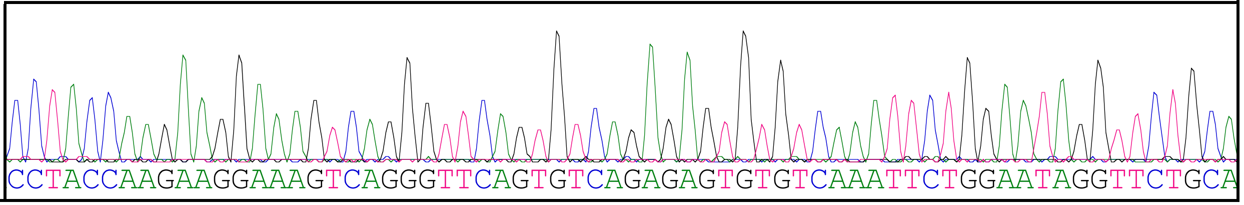

1330 1340 1350 1360 1370 1380

GAAATAGATAGCTCCCAAATTATAACCAGCCCTCTTCCTCCAGTGTCTTCCCCTCCTCCTGCATCT

A6907 PIK3C3.SEQ(1>2676) → gaaatagatagctcccaaattataaccagccctcttcctccagtgtcttcccctcctcctgcatct

140419R9088-1R22(A6907)A6907F2_Z_D01.ab1(1>700) →

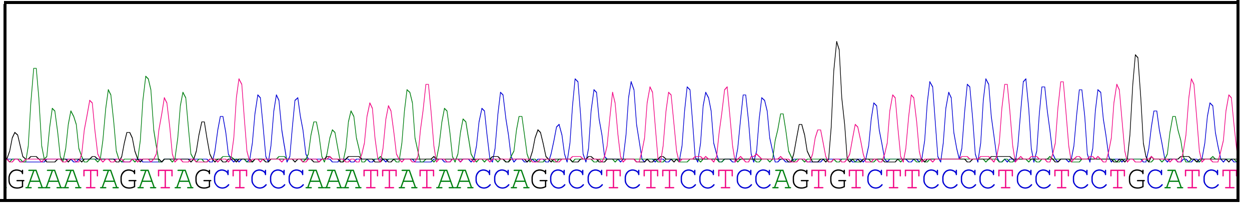

1390 1400 1410 1420 1430 1440 1450

AAAACAAAAGAAAGTTCAGACGGTGAAAGTCTGGAACAAGATCTGTGTACCTTCCTGATATCAAGA

A6907 PIK3C3.SEQ(1>2676) → aaaacaaaagaaagttcagacggtgaaagtctggaacaagatctgtgtaccttcctgatatcaaga
140419R9088-1R22(A6907)A6907F2_Z_D01.ab1(1>700) →

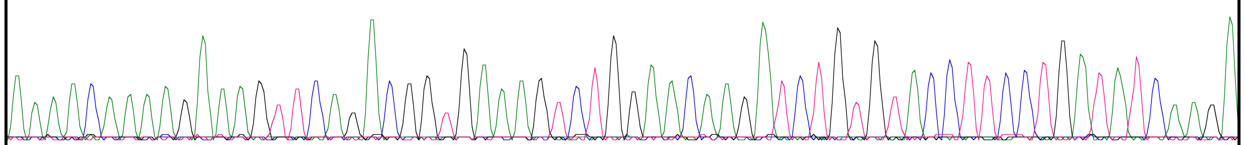

AAAACAAAAGAAAGTTCAGACGGTGAAAGTCTGGAACAAGATCTGTGTACCTTCCTGATATCAAGA

1460 1470 1480 1490 1500 1510

GCCTGCAAAAACTCAACACTGGCTAATTATTTATACTGGTATGTGATAGTAGAATGTGAAGGTCAA

A6907 PIK3C3.SEQ(1>2676) → gcctgcaaaaactcaacactggctaattatttatactggtatgtgatagtagaatgtgaaggtcaa
140419R9088-1R22(A6907)A6907F2_Z_D01.ab1(1>700) →

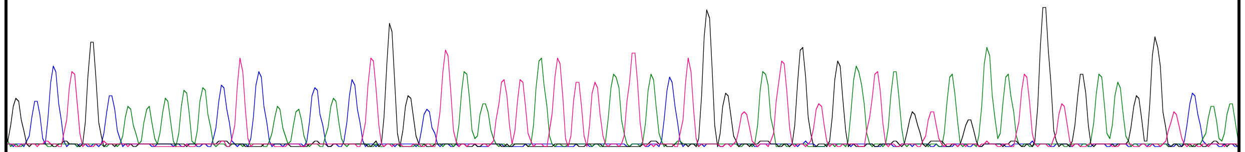

GCCTGCAAAAACTCAACACTGGCTAATTATTTATACTGGTATGTGATAGTAGAATGTGAAGGTCAA

1520 1530 1540 1550 1560 1570 1580

GATACTCAGCAGAGAGATCCAAAGACCCATGAGATGTACTTGAATGTAATGAGACGATTCAGCCAA

A6907 PIK3C3.SEQ(1>2676) → gatactcagcagagagatccaaagacccatgagatgtacttgaatgtaatgagacgattcagccaa
140419R9088-1R22(A6907)A6907F2_Z_D01.ab1(1>700) →

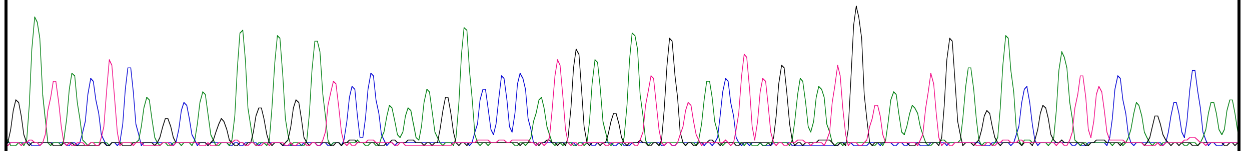

GATACTCAGCAGAGAGATCCAAAGACCCATGAGATGTACTTGAATGTAATGAGACGATTCAGCCAA

1590 1600 1610 1620 1630 1640 165

GCATTGTTGAAGGGTGATAAATCTGTCAGAGTTATGCGTTCGTTGCTGGCTGCACAGCAGACATTT

A6907 PIK3C3.SEQ(1>2676) → gcattgttgaagggtgataaatctgtcagagttatgcgttcgttgctggctgcacagcagacattt

1590 1600 1610 1620 1630 1640 1650

GCATTGTTGAAGGGTGATAAATCTGTCAGAGTTATGCGTTCGTTGCTGGCTGCACAGCAGACATTT

140419R9088-1R22(A6907)A6907F2_Z_D01.ab1(1>700)→

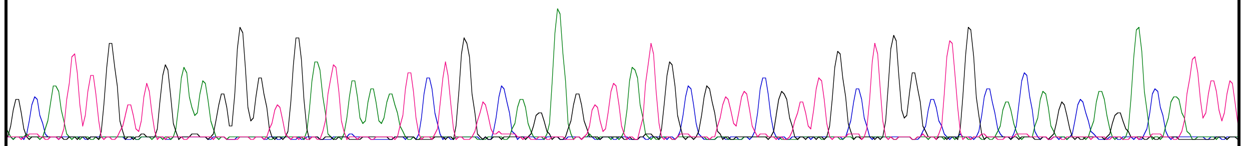

GCATTGTTGAAGGGTGATAAATCTGTCAGAGTTATGCGTTCGTTGCTGGCTGCACAGCAGACATTT

140419R9088-1R22(A6907)A6907F3_Z_E01.ab1(1>700)→

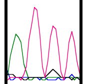

ATTT

1660 1670 1680 1690 1700 1710

GTAGATCGACTGGTGCACCTAATGAAGGCAGTTCAACGTGAAAGTGGGAATCGTAAGAAAAAGAAT

A6907 PIK3C3.SEQ(1>2676) → gtagatcgactggtgcacctaatgaaggcagttcaacgtgaaagtgggaatcgtaagaaaaagaat

140419R9088-1R22(A6907)A6907F2_Z_D01.ab1(1>700)→

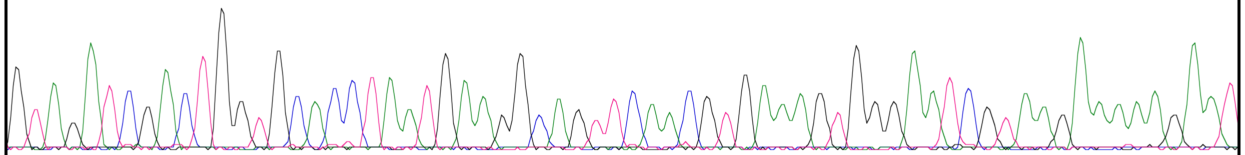

GTAGATCGACTGGTGCACCTAATGAAGGCAGTTCAACGTGAAAGTGGGAATCGTAAGAAAAAGAAT

140419R9088-1R22(A6907)A6907F3_Z_E01.ab1(1>700)→

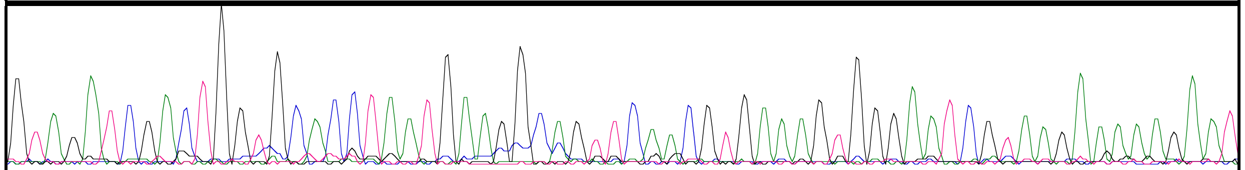

GTAGATCGACTGGTGCACCTAATGAAGGCAGTTCAACGTGAAAGTGGGAATCGTAAGAAAAAGAAT

1720 1730 1740 1750 1760 1770 1780

GAGAGACTACAGGCATTGCTTGGAGACAATGAAAAGATGAACTTGTCAGATGTGGAACTTATCCCA

A6907 PIK3C3.SEQ(1>2676) → gagagactacaggcattgcttggagacaatgaaaagatgaacttgtcagatgtggaacttatccca

140419R9088-1R22(A6907)A6907F2_Z_D01.ab1(1>700)→

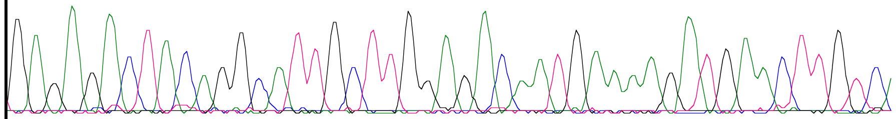

GAGAGACTACAGGCATTGCTTGGAGACAATGAAAAGATGAACTTGTC

1720 1730 1740 1750 1760 1770 1780

GAGAGACTACAGGCATTGCTTGGAGACAATGAAAAGATGAACTTGTCAGATGTGGAACTTATCCCA

140419R9088-1R22(A6907)A6907F3_Z_E01.ab1(1>700) →

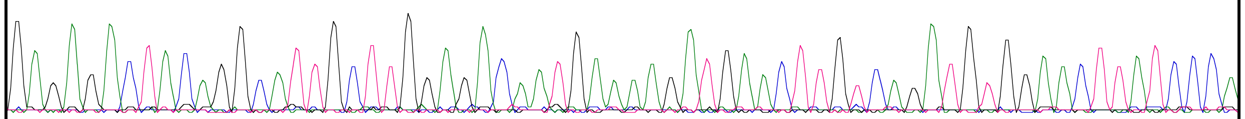

GAGAGACTACAGGCATTGCTTGGAGACAATGAAAAGATGAACTTGTCAGATGTGGAACTTATCCCA

1790 1800 1810 1820 1830 1840

TTGCCTTTAGAACCCCAGGTGAAAATTAGAGGAATAATCCCAGAAACAGCTACATTATTCAAGAGT

A6907 PIK3C3.SEQ(1>2676) → ttgcctttagaaccccaggtgaaaattagaggaataatcccagaaacagctacattattcaagagt

140419R9088-1R22(A6907)A6907F3_Z_E01.ab1(1>700) →

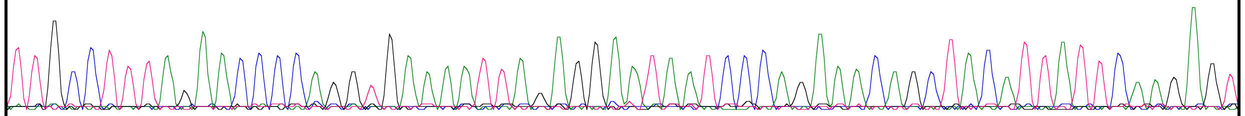

TTGCCTTTAGAACCCCAGGTGAAAATTAGAGGAATAATCCCAGAAACAGCTACATTATTCAAGAGT

1850 1860 1870 1880 1890 1900 1910

GCCCTTATGCCTGCACAGTTATTCTTTAAGACAGAAGATGGAGGCAAATACCCAGTTATATTTAAA

A6907 PIK3C3.SEQ(1>2676) → gcccttatgcctgcacagttattctttaagacagaagatggaggcaaatacccagttatatttaaa

140419R9088-1R22(A6907)A6907F3_Z_E01.ab1(1>700) →

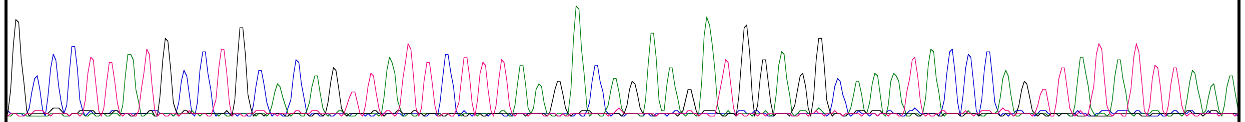

GCCCTTATGCCTGCACAGTTATTCTTTAAGACAGAAGATGGAGGCAAATACCCAGTTATATTTAAA

1920 1930 1940 1950 1960 1970 1980

CATGGGGATGATTTACGCCAAGATCAACTTATTCTCCAAATCATTTCACTCATGGACAAGCTGTTG

A6907 PIK3C3.SEQ(1>2676) → catggggatgatttacgccaagatcaacttattctccaaatcatttcactcatggacaagctgttg

140419R9088-1R22(A6907)A6907F3_Z_E01.ab1(1>700) →

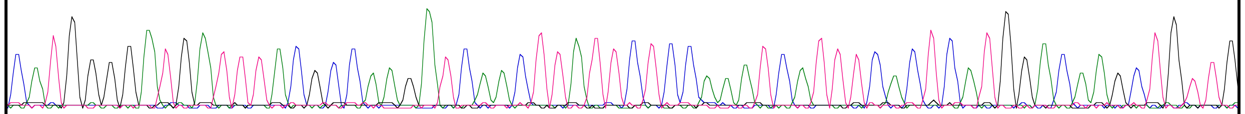

CATGGGGATGATTTACGCCAAGATCAACTTATTCTCCAAATCATTTCACTCATGGACAAGCTGTTG

1990 2000 2010 2020 2030 2040

CGGAAAGAAAATCTGGATTTAAAGTTGACACCCTATAAAGTATTAGCCACTAGTACAAAACATGGC

A6907 PIK3C3.SEQ(1>2676) → cggaaagaaaatctggatttaaagttgacaccctataaagtattagccactagtacaaaacatggc

140419R9088-1R22(A6907)A6907F3_Z_E01.ab1(1>700) →

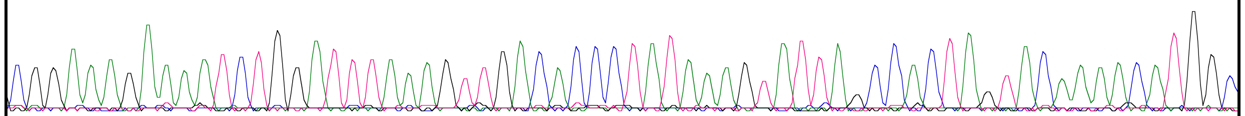

CGGAAAGAAAATCTGGATTTAAAGTTGACACCCTATAAAGTATTAGCCACTAGTACAAAACATGGC

140419R9088-1R22(A6907)M13-_Z_B01.ab1(1>700) ←

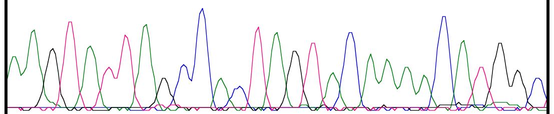

AAGTATTAGCCACTAGTACAAAACATGGC

2050 2060 2070 2080 2090 2100 2110

TTCATGCAGTTTATCCAATCAGTTCCTGTTGCTGAAGTTCTTGATACAGAAGGAAGCATTCAGAAT

A6907 PIK3C3.SEQ(1>2676) → ttcatgcagtttatccaatcagttcctgttgctgaagttcttgatacagaaggaagcattcagaat

140419R9088-1R22(A6907)A6907F3_Z_E01.ab1(1>700) →

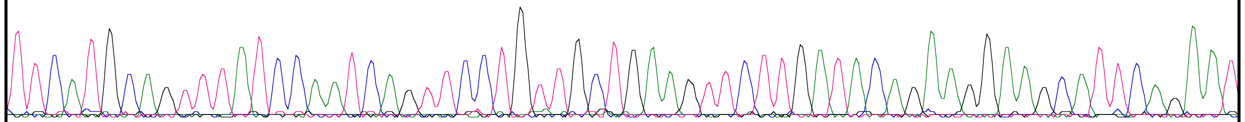

TTCATGCAGTTTATCCAATCAGTTCCTGTTGCTGAAGTTCTTGATACAGAAGGAAGCATTCAGAAT

140419R9088-1R22(A6907)M13-_Z_B01.ab1(1>700) ←

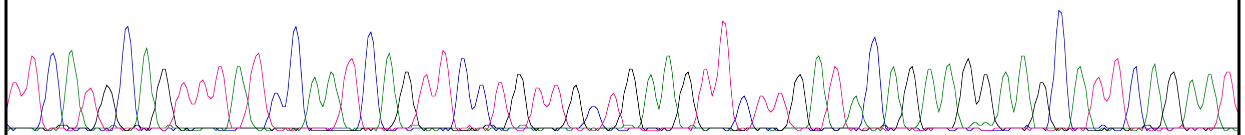

TTCATGCAGTTTATCCAATCAGTTCCTGTTGCTGAAGTTCTTGATACAGAAGGAAGCATTCAGAAT

2120 2130 2140 2150 2160 2170

TTTTTTAGAAAATATGCACCAAGTGAGAATGGTCCGAATGGAATCAGTGCTGAAGTTATGGATACT

A6907 PIK3C3.SEQ(1>2676) → tttttagaaaatatgcaccaagtgagaatggtccgaatggaatcagtgctgaagttatggatact

2120 2130 2140 2150 2160 2170

TTTTTTAGAAAATATGCACCAAGTGAGAATGGTCCGAATGGAATCAGTGCTGAAGTTATGGATACT

140419R9088-1R22(A6907)A6907F3_Z_E01.ab1(1>700) →

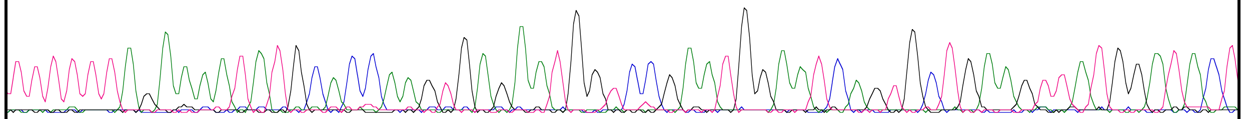

TTTTTTAGAAAATATGCACCAAGTGAGAATGGTCCGAATGGAATCAGTGCTGAAGTTATGGATACT

140419R9088-1R22(A6907)M13-_Z_B01.ab1(1>700) ←

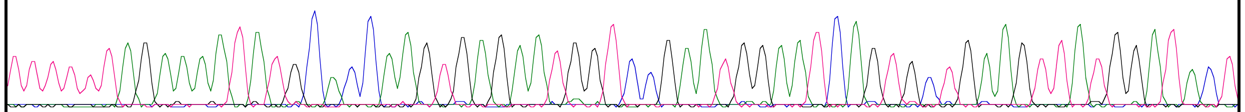

TTTTTTAGAAAATATGCACCAAGTGAGAATGGTCCGAATGGAATCAGTGCTGAAGTTATGGATACT

2180 2190 2200 2210 2220 2230 2240

TATGTTAAAAGCTGTGCTGGATATTGCGTGATTACATATATTCTTGGAGTTGGGGACAGGCACCTG

A6907 PIK3C3.SEQ(1>2676) → tatgttaaaagctgtgctggatattgcgtgattacatatattcttggagttggggacaggcacctg

140419R9088-1R22(A6907)A6907F3_Z_E01.ab1(1>700) →

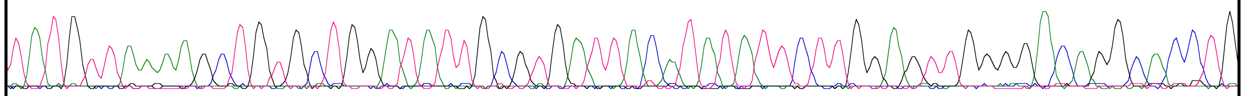

TATGTTAAAAGCTGTGCTGGATATTGCGTGATTACATATATTCTTGGAGTTGGGGACAGGCACCTG

140419R9088-1R22(A6907)M13-_Z_B01.ab1(1>700) ←

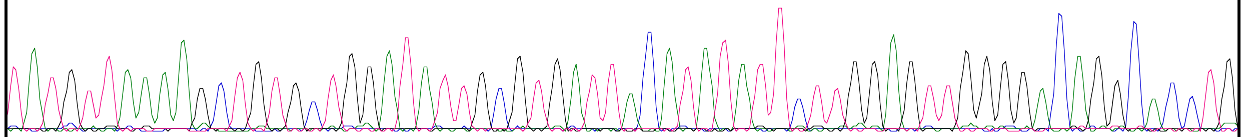

TATGTTAAAAGCTGTGCTGGATATTGCGTGATTACATATATTCTTGGAGTTGGGGACAGGCACCTG

2250 2260 2270 2280 2290 2300 2310

GATAATCTTTTGCTGACGAAAACAGGCAAACTCTTCCACATAGACTTTGGATATATTTTGGGTAGG

A6907 PIK3C3.SEQ(1>2676) → gataatcttttgctgacgaaaacaggcaaactcttccacatagactttggatatattttgggtagg

140419R9088-1R22(A6907)A6907F3_Z_E01.ab1(1>700) →

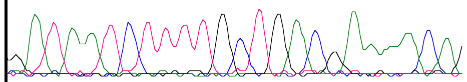

GATAATCTTTTGCTGACGAAAACA

2250 2260 2270 2280 2290 2300 231[illegible]

GATAATCTTTTGCTGACGAAAACAGGCAAACTCTTCCACATAGACTTTGGATATATTTTGGGTAGG

140419R9088-1R22(A6907)M13-_Z_B01.ab1(1>700) ←

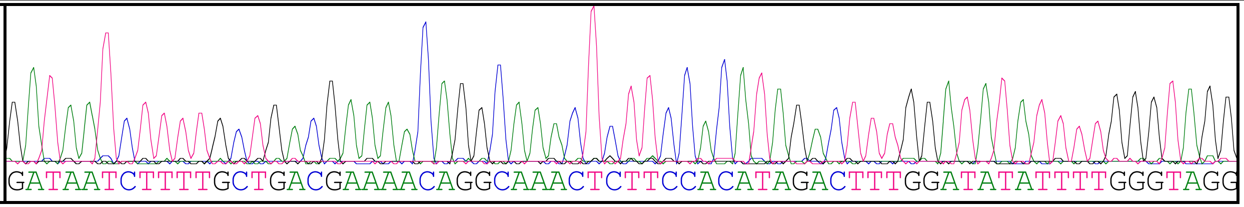

2320 2330 2340 2350 2360 2370

GACCCAAAGCCTCTTCCTCCTCCAATGAAGCTGAATAAAGAAATGGTAGAAGGAATGGGCGGCACA

A6907 PIK3C3.SEQ(1>2676) → gacccaaagcctcttcctcctccaatgaagctgaataaagaaatggtagaaggaatgggcggcaca

140419R9088-1R22(A6907)M13-_Z_B01.ab1(1>700) ←

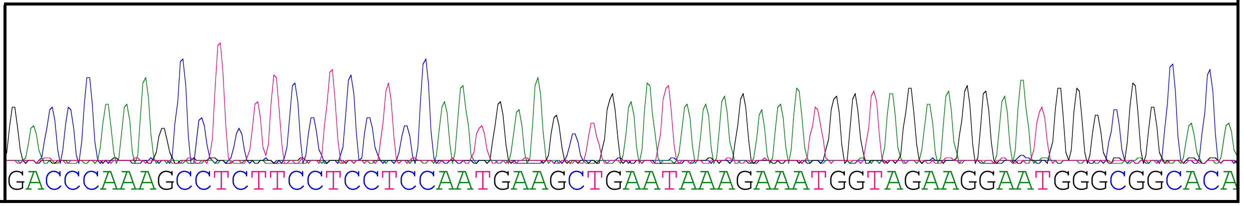

2380 2390 2400 2410 2420 2430 2440

CAGAGCGAACAATACCAAGAGTTCCGTAAACAGTGCTACACAGCTTTTCTCCACCTTCGAAGGTAT

A6907 PIK3C3.SEQ(1>2676) → cagagcgaacaataccaagagttccgtaaacagtgctacacagcttttctccaccttcgaaggtat

140419R9088-1R22(A6907)M13-_Z_B01.ab1(1>700) ←

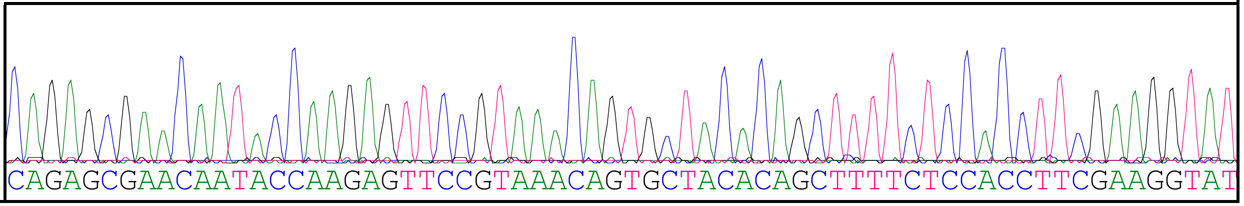

2450 2460 2470 2480 2490 2500

TCTAACCTTATTTTGAACCTGTTTTCCTTGATGGTGGATGCAAACATTCCAGATATTGCTCTTGAA

A6907 PIK3C3.SEQ(1>2676) → tctaaccttattttgaacctgttttccttgatggtggatgcaaacattccagatattgctcttgaa

140419R9088-1R22(A6907)M13-_Z_B01.ab1(1>700) ←

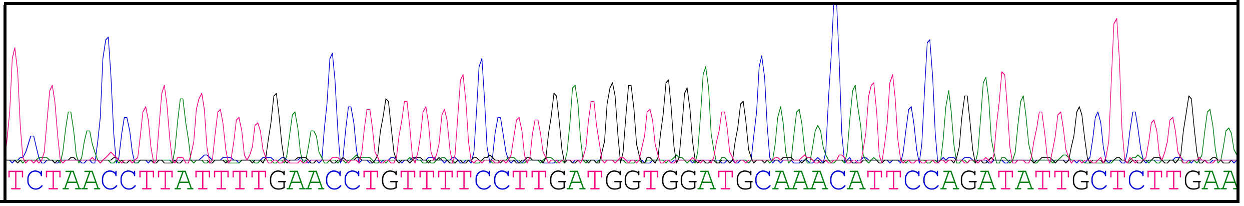

2510 2520 2530 2540 2550 2560 2570

CCAGATAAAACTGTGAAAAAGGTTCAGGATAAATTTCGTCTAGACCTGTCCGATGAAGAGGCAGTC

A6907 PIK3C3.SEQ(1>2676) → ccagataaaactgtgaaaaaggttcaggataaatttcgtctagacctgtccgatgaagaggcagtc

140419R9088-1R22(A6907)M13-_Z_B01.ab1(1>700) ←

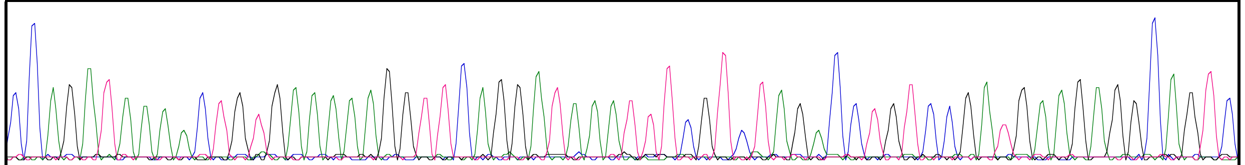

CCAGATAAAACTGTGAAAAAGGTTCAGGATAAATTTCGTCTAGACCTGTCCGATGAAGAGGCAGTC

2580 2590 2600 2610 2620 2630 2640

CATTACATGCAGAGTCTGATTGATGAAAGTGTCCATGCTCTGTTTGCTGCGGTGGTAGAACAGATT

A6907 PIK3C3.SEQ(1>2676) → cattacatgcagagtctgattgatgaaagtgtccatgctctgtttgctgcggtggtagaacagatt

140419R9088-1R22(A6907)M13-_Z_B01.ab1(1>700) ←

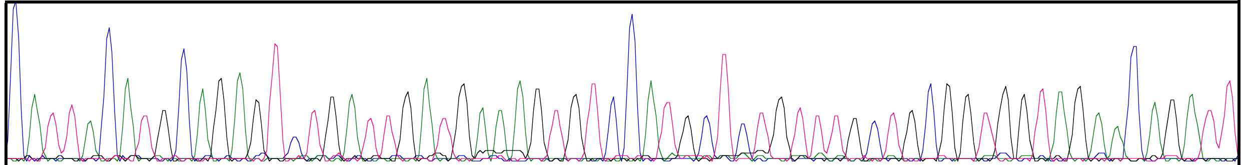

CATTACATGCAGAGTCTGATTGATGAAAGTGTCCATGCTCTGTTTGCTGCGGTGGTAGAACAGATT

2650 2660 2670 2680 2690 2700

CATAAGTTCGCCCAGTACTGGAGAAAATGAGGATCCCGGGCCCGTCGACTGCAGAGGCCTGCATGC

A6907 PIK3C3.SEQ(1>2676) → cataagttcgcccagtactggagaaaatgaggatcc

140419R9088-1R22(A6907)M13-_Z_B01.ab1(1>700) ←

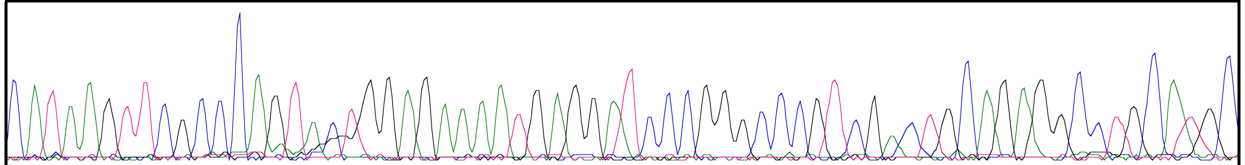

CATAAGTTCGCCCAGTACTGGAGAAAATGAGGATCCCGGGCCCGTCGACTGCAGAGGCCTGCATGC

2710

AAGCTTGGCGT

140419R9088-1R22(A6907)M13-_Z_B01.ab1(1>700) ←

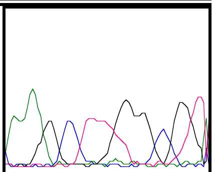

AAGCTTGGCGT

Supplementary materials 2 pPIK-IRES2-AcGFP1-EN sequence

ORIGIN

1 TAGTTATGTC GACAGTATGC TACAGTCACT TAAGCACAAG CGACAGTATA TGAACTAAAA
61 GAGAGAATAT ATGCAATGGT TGGCATTTAA CCATCAAAAC AAATCATACA ATAAAAAGTT
121 TTTATGATTT CCAGAGTTTT TGAACGAGGA GATCAAATTC CATTTATGTT CATATATATT
181 ACAACATATG CAGGTAAATG AAAGCAATTC TCCTTGTGTT CTGGTGAATT AAAGGAGTAT
241 GCTTTAAAGT CTATTTCTTT ACAATTTTAC TTAATATTTA CAGAAAAATC TATATGTAGT
301 ATTGATAAGA TGTAGGATTG TTATATACCA TTATTTGAGT CATCCTTAAA CACTTGAATT
361 ATATTGTATG ATAGCATACT TGGTAAGAGA AGATTCCACA AAAATAGGGA TGGTACACCA
421 TATGCAAGTT CCCATTCCTA TTCTGATTGA TACAGTACAT TGACAATTCA TGCCAATGGT
481 GCTAATTCAA TAGGCTGAAT GGCTGGTGTT ATCAGGTTTA TCAAATAAAA GCATTCAGTA
541 AAGTAATGGT TCTCCTTTCT TCAGGTGCAT TTTCATACTC TTTCAAATGG AGAATGGATT
601 TTCTTTAATG AAAGAAAAAT CATTTTTCTA GAGCTCTGCA TTCAATTCTG TAGCATACTT
661 GGAGAAACTG CATTTAAAAG GCAGCCAAAA AGTATTCATT TTTATCAAAA TTTCAAAATT
721 ATAGCCTGCC TTTGCAACAC TGCAGTTTTT ATGATAAAAT CATGGCAATG ACTGATTCTA
781 TCAATATTGT ATAAAAAGAC TTTGAAACAA TTGCATTTAT ATAATATGTA AACAATATTG
841 TTTTGTAAAT AAATGTCTCC TTTTTTATTT ACTTTGGTAT ATTTTTATGT AAGGATATTT
901 CAAATTAAGT ATTAAGGCAC AAATACATGT CATGTGACAG AAAAGCAAAT GCTTATATTT
961 CGGAGCAAAT TAGCTGATTA AATAGTGGTC TTAAAACTCC ATATGCTAAT GGTTAGATGG
1021 TTATATTACA ATCATTTTAT ATTTTTTTAC ATTATTAACA TTCACTATGG ATTCATGATG
1081 GCTGTATAAT GTGAATGTGA AATTTCAATG GTTTACTGTC AATGTATTCA AATCTCAACG
1141 TTCCATTATT TTAATACTTA TAAGTAAGCA TACCAAAATG ATTTAACTCA ATTATCTGAA
1201 ATCAGAATAA TAAACTGATG ATATCTTAAG AATTGTTAAT TTAATTTTAT AATTCGATAA
1261 TGAATATATT TCTCCATATA TTTACTTCTA TTTTGTAAAT TAGGATTTTG TTAATCAAAT
1321 ACATTGTACT TATGACTAAG TGAAATTATT TCTTACATCT AATGTGTAGA AACAATATAA
1381 ATTATATTAA AGTGTTTTCA CCTTTTTTGA AAGACACAAC AGTTTTATGT TATAATGATT
1441 AATTCTGAAT TTTTGGTTTT CATTTTATTA TAACAGTTTA ATGATTTAGC ATTGAAGTTT
1501 GGTTTCAAAA TTTTAAGTCT GCTACTATAG TGAGACTCCT GATAGTAATC AATTACGGGG
1561 TCATTAGTTC ATAGCCCATA TATGGAGTTC CGCGTTACAT AACTTACGGT AAATGGCCCG
1621 CCTGGCTGAC CGCCCAACGA CCCCCGCCCA TTGACGTCAA TAATGACGTA TGTTCCCATA
1681 GTAACGCCAA TAGGGACTTT CCATTGACGT CAATGGGTGG AGTATTTACG GTAAACTGCC
1741 CACTTGGCAG TACATCAAGT GTATCATATG CCAAGTACGC CCCCTATTGA CGTCAATGAC
1801 GGTAAATGGC CCGCCTGGCA TTATGCCCAG TACATGACCT TATGGGACTT TCCTACTTGG
1861 CAGTACATCT ACGTATTAGT CATCGCTATT ACCATGGTGA TGCGGTTTTG GCAGTACATC
1921 AATGGGCGTG GATAGCGGTT TGACTCACGG GGATTTCCAA GTCTCCACCC CATTGACGTC
1981 AATGGGAGTT TGTTTTGGCA CCAAAATCAA CGGGACTTTC CAAAATGTCG TAACAACTCC
2041 GCCCCATTGA CGCAAATGGG CGGTAGGCGT GTACGGTGGG AGGTCTATAT AAGCAGAGCT
2101 GGTTTAGTGA ACCGTCAGAT CCGCTAGCGC TACCGGACTC AGATCTCGAG ATGGGGGAAG
2161 CAGAGAAGTT TCACTACATC TACAGTTGTG ACCTGGATAT CAACGTGCAG CTTAAGATAG
2221 GAAGTTTGGA AGGGAAGAGA GAACAAAAGA GTTATAAAGC CGTTCTAGAA GACCCAATGT
2281 TGAAGTTTTC AGGACTATAC CAAGAAACAT GTTCTGACCT CTATGTCACT TGCCAAGTTT
2341 TTGCAGAAGG AAAGCCTCTG GCCTTGCCAG TAAGAACTTC GTATAAGGCA TTTAGCACAA
2401 GATGGAACTG GAATGAATGG CTGAAACTGC CTGTGAAATA CCCTGACCTG CCCAGGAATG
2461 CCCAAGTGGC CCTGACTATA TGGGATGTCT ATGGACCAGG AAAGGCAGTG CCTGTGGGTG
2521 GAACAACAGT TTCATTGTTT GGAAAATACG GCATGTTTCG CCAAGGGATG CATGACTTGA

2581 AAGTCTGGCC TAATGTGGAA GCAGACGGAT CAGAACCCAC AAAAACTCCT GGCAGAACAA
2641 GCAGTACTCT CTCAGAAGAT CAGATGAGCC GTCTTGCCAA GCTCACCAAG GCTCATCGAC
2701 AAGGTCACAT GGTGAAAGTA GATTGGCTGG ACAGACTGAC CTTTAGAGAA ATAGAAATGA
2761 TAAATGAGAG TGAAAAACGA AGTTCTAATT TCATGTACTT GATGGTTGAG TTTCGATGTG
2821 TCAAGTGTGA TGATAAAGAA TATGGTATTG TTTATTATGA AAAGGATGGT GACGAGTCAT
2881 CTCCCATTTT AACGGGCTTT GAGATAGTGA AAGTTCCTGA CCCTCAAATG TCTATGGAGA
2941 ATTTAGTTGA GAGCAAACAC CACAAACTTG CTCGGAGTTT AAGAAGTGGA CCTTCTGACC
3001 ATGGTCTCAA ACCTAATGCT GCCACGAGAG ATCAACTGAA TATTATTGTG AGTTATCCAC
3061 CAACCAAGCA ACTTACATAT GAAGAACAAG ATCTTGTATG GAAGTTTAGA TATTATCTTA
3121 CTAATCAAGA AAAAGCTTTG ACAAAGTTCT TGAAATGCGT TAATTGGGAT CTACCTCAGG
3181 AGGCCAAACA GGCATTGGAA CTTCTGGGAA AATGGAAGCC AATGGATGTA GAGGATTCCT
3241 TGGAGCTATT ATCATCCCAT TACGCCAATC CAACAGTGAG ACGTTATGCA GTTGCCCGAT
3301 TACGGCAGGC AGATGATGAG GATTTGTTGA TGTACCTATT ACAGCTCGTC CAAGCTCTCA
3361 AATATGAAAA TTTTGATGAC ATAAAGAATG GATTAGAACC TACCAAGAAG GAAAGTCAGG
3421 GTTCAGTGTC AGAGAGTGTG TCAAATTCTG GAATAGGTTC TGCAGAAATA GATAGCTCCC
3481 AAATTATAAC CAGCCCTCTT CCTCCAGTGT CTTCCCCTCC TCCTGCATCT AAAACAAAAG
3541 AAAGTTCAGA CGGTGAAAGT CTGGAACAAG ATCTGTGTAC CTTCCTGATA TCAAGAGCCT
3601 GCAAAAACTC AACACTGGCT AATTATTTAT ACTGGTATGT GATAGTAGAA TGTGAAGGTC
3661 AAGATACTCA GCAGAGAGAT CCAAAGACCC ATGAGATGTA CTTGAATGTA ATGAGACGAT
3721 TCAGCCAAGC ATTGTTGAAG GGTGATAAAT CTGTCAGAGT TATGCGTTCG TTGCTGGCTG
3781 CACAGCAGAC ATTTGTAGAT CGACTGGTGC ACCTAATGAA GGCAGTTCAA CGTGAAAGTG
3841 GGAATCGTAA GAAAAAGAAT GAGAGACTAC AGGCATTGCT TGGAGACAAT GAAAAGATGA
3901 ACTTGTCAGA TGTGGAACTT ATCCCATTGC CTTTAGAACC CCAGGTGAAA ATTAGAGGAA
3961 TAATCCCAGA AACAGCTACA TTATTCAAGA GTGCCCTTAT GCCTGCACAG TTATTCTTTA
4021 AGACAGAAGA TGGAGGCAAA TACCCAGTTA TATTTAAACA TGGGGATGAT TTACGCCAAG
4081 ATCAACTTAT TCTCCAAATC ATTTCACTCA TGGACAAGCT GTTGCGGAAA GAAAATCTGG
4141 ATTTAAAGTT GACACCCTAT AAAGTATTAG CCACTAGTAC AAAACATGGC TTCATGCAGT
4201 TTATCCAATC AGTTCCTGTT GCTGAAGTTC TTGATACAGA AGGAAGCATT CAGAATTTTT
4261 TTAGAAAATA TGCACCAAGT GAGAATGGTC CGAATGGAAT CAGTGCTGAA GTTATGGATA
4321 CTTATGTTAA AAGCTGTGCT GGATATTGCG TGATTACATA TATTCTTGGA GTTGGGGACA
4381 GGCACCTGGA TAATCTTTTG CTGACGAAAA CAGGCAAACT CTTCCACATA GACTTTGGAT
4441 ATATTTTGGG TAGGGACCCA AAGCCTCTTC CTCCTCCAAT GAAGCTGAAT AAAGAAATGG
4501 TAGAAGGAAT GGGCGGCACA CAGAGCGAAC AATACCAAGA GTTCCGTAAA CAGTGCTACA
4561 CAGCTTTTCT CCACCTTCGA AGGTATTCTA ACCTTATTTT GAACCTGTTT TCCTTGATGG
4621 TGGATGCAAA CATTCCAGAT ATTGCTCTTG AACCAGATAA AACTGTGAAA AAGGTTCAGG
4681 ATAAATTTCG TCTAGACCTG TCCGATGAAG AGGCAGTCCA TTACATGCAG AGTCTGATTG
4741 ATGAAAGTGT CCATGCTCTG TTTGCTGCGG TGGTAGAACA GATTCATAAG TTCGCCCAGT
4801 ACTGGAGAAA ATGA(PIK3C3)GGATCC GCCCCTCTCC CTCCCCCCCC CCTAACGTTA CTGGCCGAAG
4861 CCGCTTGGAA TAAGGCCGGT GTGCGTTTGT CTATATGTTA TTTTCCACCA TATTGCCGTC
4921 TTTTGGCAAT GTGAGGGCCC GGAAACCTGG CCCTGTCTTC TTGACGAGCA TTCCTAGGGG
4981 TCTTTCCCCT CTCGCCAAAG GAATGCAAGG TCTGTTGAAT GTCGTGAAGG AAGCAGTTCC
5041 TCTGGAAGCT TCTTGAAGAC AAACAACGTC TGTAGCGACC CTTTGCAGGC AGCGGAACCC
5101 CCCACCTGGC GACAGGTGCC TCTGCGGCCA AAAGCCACGT GTATAAGATA CACCTGCAAA
5161 GGCGGCACAA CCCCAGTGCC ACGTTGTGAG TTGGATAGTT GTGGAAAGAG TCAAATGGCT

5221 CTCCTCAAGC GTATTCAACA AGGGGCTGAA GGATGCCCAG AAGGTACCCC ATTGTATGGG
5281 ATCTGATCTG GGGCCTCGGT ACACATGCTT TACATGTGTT TAGTCGAGGT TAAAAAAACG
5341 TCTAGGCCCC CCGAACCACG GGGACGTGGT TTTCCTTTGA AAAACACGAT GATAATATGG
5401 CCACAACCAT GGTGAGCAAG GGCGCCGAGC TGTTCACCGG CATCGTGCCC ATCCTGATCG
5461 AGCTGAATGG CGATGTGAAT GGCCACAAGT TCAGCGTGAG CGGCGAGGGC GAGGGCGATG
5521 CCACCTACGG CAAGCTGACC CTGAAGTTCA TCTGCACCAC CGGCAAGCTG CCTGTGCCCT
5581 GGCCCACCCT GGTGACCACC CTGAGCTACG GCGTGCAGTG CTTCTCACGC TACCCCGATC
5641 ACATGAAGCA GCACGACTTC TTCAAGAGCG CCATGCCTGA GGGCTACATC CAGGAGCGCA
5701 CCATCTTCTT CGAGGATGAC GGCAACTACA AGTCGCGCGC CGAGGTGAAG TTCGAGGGCG
5761 ATACCCTGGT GAATCGCATC GAGCTGACCG GCACCGATTT CAAGGAGGAT GGCAACATCC
5821 TGGGCAATAA GATGGAGTAC AACTACAACG CCCACAATGT GTACATCATG ACCGACAAGG
5881 CCAAGAATGG CATCAAGGTG AACTTCAAGA TCCGCCACAA CATCGAGGAT GGCAGCGTGC
5941 AGCTGGCCGA CCACTACCAG CAGAATACCC CCATCGGCGA TGGCCCTGTG CTGCTGCCCG
6001 ATAACCACTA CCTGTCCACC CAGAGCGCCC TGTCCAAGGA CCCCAACGAG AAGCGCGATC
6061 ACATGATCTA CTTCGGCTTC GTGACCGCCG CCGCCATCAC CCACGGCATG GATGAGCTGT
6121 ACAAGTGA(GFP)GC GGCCGCGACT CTAGATCATA ATCAGCCATA CCACATTTGT AGAGGTTTTA
6181 CTTGCTTTAA AAAACCTCCC ACACCTCCCC CTGAACCTGA AACATAAAAT GAATGCAATT
6241 GTTGTTGTTA ACTTGTTTAT TGCAGCTTAT AATGGTTACA AATAAAGCAA TAGCATCACA
6301 AATTTCACAA ATAAAGCATT TTTTTCACTG CATTCTAGTT GTGGTTTGTC CAAACTCATC
6361 AATGTATCTT AAGGCGTAAA TTGTAAGCGT TAATATTTTG TTAAAATTCG CGTTAAATTT
6421 TTGTTAAATC AGCTCATTTT TTAACCAATA GGCCGAAATC GGCAAAATCC CTTATAAATC
6481 AAAAGAATAG ACCGAGATAG GGTTGAGTGT TGTTCCAGTT TGGAACAAGA GTCCACTATT
6541 AAAGAACGTG GACTCCAACG TCAAAGGGCG AAAAACCGTC TATCAGGGCG ATGGCCCACT
6601 ACGTGAACCA TCACCCTAAT CAAGTTTTTT GGGGTCGAGG TGCCGTAAAG CACTAAATCG
6661 GAACCCTAAA GGGAGCCCCC GATTTAGAGC TTGACGGGGA AAGCCGGCGA ACGTGGCGAG
6721 AAAGGAAGGG AAGAAAGCGA AAGGAGCGGG CGCTAGGGCG CTGGCAAGTG TAGCGGTCAC
6781 GCTGCGCGTA ACCACCACAC CCGCCGCGCT TAATGCGCCG CTACAGGGCG CGTCAGGTGG
6841 CACTTTTCGG GGAAATGTGC GCGGAACCCC TATTTGTTTA TTTTTCTAAA TACATTCAAA
6901 TATGTATCCG CTCATGAGAC AATAACCCTG ATAAATGCTT CAATAATATT GAAAAAGGAA
6961 GAGTCCTGAG GCGGAAAGAA CCAGCTGTGG AATGTGTGTC AGTTAGGGTG TGGAAAGTCC
7021 CCAGGCTCCC CAGCAGGCAG AAGTATGCAA AGCATGCATC TCAATTAGTC AGCAACCAGG
7081 TGTGGAAAGT CCCCAGGCTC CCCAGCAGGC AGAAGTATGC AAAGCATGCA TCTCAATTAG
7141 TCAGCAACCA TAGTCCCGCC CCTAACTCCG CCCATCCCGC CCCTAACTCC GCCCAGTTCC
7201 GCCCATTCTC CGCCCCATGG CTGACTAATT TTTTTTATTT ATGCAGAGGC CGAGGCCGCC
7261 TCGGCCTCTG AGCTATTCCA GAAGTAGTGA GGAGGCTTTT TTGGAGGCCT AGGCTTTTGC
7321 AAAGATCGAT CAAGAGACAG GATGAGGATC GTTTCGCATG ATTGAACAAG ATGGATTGCA
7381 CGCAGGTTCT CCGGCCGCTT GGGTGGAGAG GCTATTCGGC TATGACTGGG CACAACAGAC
7441 AATCGGCTGC TCTGATGCCG CCGTGTTCCG GCTGTCAGCG CAGGGGCGCC CGGTTCTTTT
7501 TGTCAAGACC GACCTGTCCG GTGCCCTGAA TGAACTGCAA GACGAGGCAG CGCGGCTATC
7561 GTGGCTGGCC ACGACGGGCG TTCCTTGCGC AGCTGTGCTC GACGTTGTCA CTGAAGCGGG
7621 AAGGGACTGG CTGCTATTGG GCGAAGTGCC GGGGCAGGAT CTCCTGTCAT CTCACCTTGC
7681 TCCTGCCGAG AAAGTATCCA TCATGGCTGA TGCAATGCGG CGGCTGCATA CGCTTGATCC
7741 GGCTACCTGC CCATTCGACC ACCAAGCGAA ACATCGCATC GAGCGAGCAC GTACTCGGAT
7801 GGAAGCCGGT CTTGTCGATC AGGATGATCT GGACGAAGAG CATCAGGGGC TCGCGCCAGC

7861 CGAACTGTTC GCCAGGCTCA AGGCGAGCAT GCCCGACGGC GAGGATCTCG TCGTGACCCA
7921 TGGCGATGCC TGCTTGCCGA ATATCATGGT GGAAAATGGC CGCTTTTCTG GATTCATCGA
7981 CTGTGGCCGG CTGGGTGTGG CGGACCGCTA TCAGGACATA GCGTTGGCTA CCCGTGATAT
8041 TGCTGAAGAG CTTGGCGGCG AATGGGCTGA CCGCTTCCTC GTGCTTTACG GTATCGCCGC
8101 TCCCGATTCG CAGCGCATCG CCTTCTATCG CCTTCTTGAC GAGTTCTTCT GAGCGGGACT
8161 CTGGGGTTCG AAATGACCGA CCAAGCGACG CCCAACCTGC CATCACGAGA TTTCGATTCC
8221 ACCGCCGCCT TCTATGAAAG GTTGGGCTTC GGAATCGTTT TCCGGGACGC CGGCTGGATG
8281 ATCCTCCAGC GCGGGGATCT CATGCTGGAG TTCTTCGCCC ACCCTAGGGG GAGGCTAACT
8341 GAAACACGGA AGGAGACAAT ACCGGAAGGA ACCCGCGCTA TGACGGCAAT AAAAAGACAG
8401 AATAAAACGC ACGGTGTTGG GTCGTTTGTT CATAAACGCG GGGTTCGGTC CCAGGGCTGG
8461 CACTCTGTCG ATACCCCACC GAGACCCCAT TGGGGCCAAT ACGCCCGCGT TTCTTCCTTT
8521 TCCCCACCCC ACCCCCCAAG TTCGGGTGAA GGCCCAGGGC TCGCAGCCAA CGTCGGGGCG
8581 GCAGGCCCTG CCATAGCCTC AGGTTACTCA TATATACTTT AGATTGATTT AAAACTTCAT
8641 TTTTAATTTA AAAGGATCTA GGTGAAGATC CTTTTTGATA ATCTCATGAC CAAAATCCCT
8701 TAACGTGAGT TTTCGTTCCA CTGAGCGTCA GACCCCGTAG AAAAGATCAA AGGATCTTCT
8761 TGAGATCCTT TTTTTCTGCG CGTAATCTGC TGCTTGCAAA CAAAAAAACC ACCGCTACCA
8821 GCGGTGGTTT GTTTGCCGGA TCAAGAGCTA CCAACTCTTT TTCCGAAGGT AACTGGCTTC
8881 AGCAGAGCGC AGATACCAAA TACTGTCCTT CTAGTGTAGC CGTAGTTAGG CCACCACTTC
8941 AAGAACTCTG TAGCACCGCC TACATACCTC GCTCTGCTAA TCCTGTTACC AGTGGCTGCT
9001 GCCAGTGGCG ATAAGTCGTG TCTTACCGGG TTGGACTCAA GACGATAGTT ACCGGATAAG
9061 GCGCAGCGGT CGGGCTGAAC GGGGGGTTCG TGCACACAGC CCAGCTTGGA GCGAACGACC
9121 TACACCGAAC TGAGATACCT ACAGCGTGAG CTATGAGAAA GCGCCACGCT TCCCGAAGGG
9181 AGAAAGGCGG ACAGGTATCC GGTAAGCGGC AGGGTCGGAA CAGGAGAGCG CACGAGGGAG
9241 CTTCCAGGGG GAAACGCCTG GTATCTTTAT AGTCCTGTCG GGTTTCGCCA CCTCTGACTT
9301 GAGCGTCGAT TTTTGTGATG CTCGTCAGGG GGGCGGAGCC TATGGAAAAA CGCCAGCAAC
9361 GCGGCCTTTT TACGGTTCCT GGCCTTTTGC TGGCCTTTTG CTCACATGTT CTTTCCTGCG
9421 TTATCCCCTG ATTCTGTGGA TAACCGTATT ACCGCCATGC A
